# Pre-Treatment of Rice Plants with ABA Makes Them More Tolerant to Multiple Abiotic Stress

**DOI:** 10.3390/ijms24119628

**Published:** 2023-06-01

**Authors:** Fatemeh Habibpourmehraban, Yunqi Wu, Farhad Masoomi-Aladizgeh, Ardeshir Amirkhani, Brian J. Atwell, Paul A. Haynes

**Affiliations:** 1 School of Natural Sciences, Macquarie University, North Ryde, NSW 2109, Australia; fatemeh.habibpour-mehraban@hdr.mq.edu.au (F.H.); yunqi.wu@mq.edu.au (Y.W.); farhad.masoomi-aladizgeh@mq.edu.au (F.M.-A.); ardeshir.amirkhani@mq.edu.au (A.A.); brian.atwell@mq.edu.au (B.J.A.); 2 Biomolecular Discovery Research Centre, Macquarie University, North Ryde, NSW 2109, Australia; 3 Australian Proteome Analysis Facility (APAF), Macquarie University, North Ryde, NSW 2109, Australia

**Keywords:** rice, multiple abiotic stress, proteomics, ABA pre-treatment, TCA cycle

## Abstract

Multiple abiotic stress is known as a type of environmental unfavourable condition maximizing the yield and growth gap of crops compared with the optimal condition in both natural and cultivated environments. Rice is the world’s most important staple food, and its production is limited the most by environmental unfavourable conditions. In this study, we investigated the pre-treatment of abscisic acid (ABA) on the tolerance of the IAC1131 rice genotype to multiple abiotic stress after a 4-day exposure to combined drought, salt and extreme temperature treatments. A total of 3285 proteins were identified and quantified across the four treatment groups, consisting of control and stressed plants with and without pre-treatment with ABA, with 1633 of those proteins found to be differentially abundant between groups. Compared with the control condition, pre-treatment with the ABA hormone significantly mitigated the leaf damage against combined abiotic stress at the proteome level. Furthermore, the application of exogenous ABA did not affect the proteome profile of the control plants remarkably, while the results were different in stress-exposed plants by a greater number of proteins changed in abundance, especially those which were increased. Taken together, these results suggest that exogenous ABA has a potential priming effect for enhancing the rice seedlings’ tolerance against combined abiotic stress, mainly by affecting stress-responsive mechanisms dependent on ABA signalling pathways in plants.

## 1. Introduction

Rice feeds more people than any other crop and is critical for our future food security. There are two cultivated species of rice, *Oryza sativa* (Asian rice) and *Oryza Glaberrima* (African rice), with *O. sativa* more widely grown worldwide [1]. Rice provides about 50% of the dietary caloric supply and a substantial part of the protein intake for about 520 million living people [2]. With the projected increase in the global population, rice will remain a staple.

Rice yield has increased due to technological developments which have improved farming practices and produced climate resilient high yielding cultivars. The yield is still 10–15 per cent lower than its potential, due mainly to various biotic and abiotic stresses [3]. Gradual expansion of environmental stresses as a consequence of a changing climate poses a threat to the yield of future rice production. To address this issue, changing rice production methods, producing new cultivars, and increasing the yield and productivity by developing new technologies are all being applied simultaneously. The aim of these efforts is assuring adequate supply, nutritional integrity and sustainability of rice production globally [4].

Unfavourable environmental conditions are among the major limitations to crop productivity worldwide. Abiotic stresses aggravate a variety of stresses for rice plants, mainly involving water deficiency, salinity and temperature extremes. Rice plants are mostly sensitive to various abiotic stresses [5]. Abiotic stresses, as the dominant drivers restricting agricultural growth and output of crop products annually, affect 70% of rice crops [6].

Rice crops are often subjected to parallel abiotic stresses simultaneously in field cultivation conditions, resulting in yield reductions beyond what would be expected from exposure to a single abiotic stress [7,8]. Improved tolerance to these abiotic stresses is a major research focus, aimed at producing cultivars tolerant to multiple stresses [9]. In recent years, these conditions have intensified due to the drastic fluctuations in global climate.

Abiotic stress can be the result of a severe environmental situation, causing major impairment to crop production. Susceptibility or tolerance to stress in plants is a coordinated action of generalized defences against abiotic stresses and a complex stress-specific regulatory network involving upstream signalling molecules, such as stress hormones, in crosstalk with downstream gene and protein regulation factors [10,11].

Meanwhile, research has provided significant gains in the understanding of the molecular responses of plants to environmental cues, but there is still a large gap between yields in optimal and stress conditions. Minimizing this yield gap and increasing yield stability under different stress conditions are of strategic importance in guaranteeing food for the future [12]. Proteomic technologies have become increasingly important in this area, as they are able to monitor and characterize protein profiles involved in various biological and molecular functions in plants. Many of these proteins are differentially expressed in response to a range of stress conditions [13].

Abscisic acid (ABA) is an important phytohormone, regulating various aspects of plant physiology under both stressed and non-stressed conditions [14], and basal ABA level plays an important role in plant growth and development. ABA is mainly known as a stress hormone which is responsible for controlling downstream responses to abiotic and biotic environmental changes [15,16,17,18]. The expression of ABA signalling proteins mitigates the effects of stressful conditions by adjusting the stomatal closure, osmotic regulation, growth inhibition and many other functions [19,20]. Endogenous levels of ABA in plants under harsh environmental conditions increase via ABA biosynthesis [21], yet high concentrations of ABA exogenously applied to normal plants have been reported to result in growth arrest [22]. This interesting dichotomy was one reason we chose to explore here the role of ABA over-accumulation in plant proteome responses to multiple abiotic stress conditions.

In this context, this study was conducted to elucidate the differential protein expression of the IAC1131 rice genotype exposed to a suite of multiple abiotic stress, either with or without ABA pre-treatment. IAC1131 is an upland landrace which has been shown in previous studies to be relatively tolerant to a variety of abiotic stresses [8,23,24]. The aim of this study was to investigate the effect of exogenous ABA application on plant responses to environmental stress, in addition to discovering stress-responsive proteins correlated with ABA signalling under non-stress and stress treatments and analysing the ABA-dependent and -independent pathways in rice plants under normal and stress conditions. Rice plants were subjected to multiple abiotic stress either with or without pre-treatment with ABA, and quantitative proteomic analysis was performed to identify and quantify the abundance of proteins in response to different conditions.

## 2. Results

### 2.1. Plant Growth

Plants of the IAC1131 genotype of rice were grown to the vegetative stage and, after four weeks of growth, four groups of plants including (Control − ABA), (Control + ABA), (Stress − ABA) and (Stress + ABA), were used for proteome analysis at the molecular level.

### 2.2. Proteome Profile of IAC1131 Rice Plants in Different Treatment Conditions

**Figure 1:**
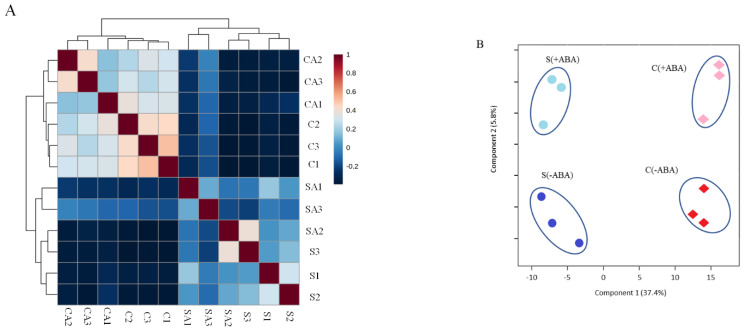
Proteomics profiling of IAC1131 genotype samples under control (Control − ABA, Control + ABA) and multiple abiotic stress conditions (Stress − ABA, Stress + ABA). A total of 3285 non-redundant proteins were reproducibly identified and quantified in IAC1131 genotype samples. (**A**) Distance matrix heatmap between all samples. Pearson correlation coefficients based on protein abundance between each pair of samples are shown as a heatmap and represent the consistency between the total of proteins identified in all 12 biological replicates, three each of four samples. CA = Control + ABA, C = Control − ABA, SA = Stress + ABA, S = Stress − ABA, and numbered suffixes indicate replicate numbers. (**B**) Partial Least Square Discriminant Analysis (PLS-DA) score plot showing three biological replicates closely aligned and that 43.2% of the total variation among differentially abundant proteins could be attributed to the plant treatment condition. C(+ABA): control plants incubated with ABA are represented with a pink colour; C(−ABA): control plants non-incubated with ABA are represented with a red colour; S(+ABA): stressed plants incubated with ABA are represented with a light blue colour; S(−ABA): stressed plants non-incubated with ABA are represented with a dark blue colour.

In total, 3285 non-redundant proteins were reproducibly identified and quantified in IAC1131 rice leaf samples, at a peptide and protein FDR of less than 1%. Pearson correlation coefficients of 3285 proteins are shown as a heatmap in Figure 1A, representing the consistency among three biological replicates belonging to each of the IAC1131 samples including (Control − ABA), (Control + ABA), (Stress − ABA) and (Stress + ABA). Partial Least Square Discriminant Analysis was applied for dimensionality reduction, indicating the contribution of variables for the differences observed between the stress treatment and ABA incubation. The obtained score plot illustrated a clustering of samples by genotypes and conditions (Figure 1B). The first two components, accounting for 43.2% of the variation, were sufficient for clear separations between the control and stress conditions along component 1. Therefore, the Partial Least Square Discriminant Analysis (PLS-DA) clearly delineated the distinct expression patterns between stress and normal conditions in plants.

### 2.3. Effect of ABA Pre-Treatment on Rice Plants under Control and Multiple Abiotic Stress Conditions

**Figure 2:**
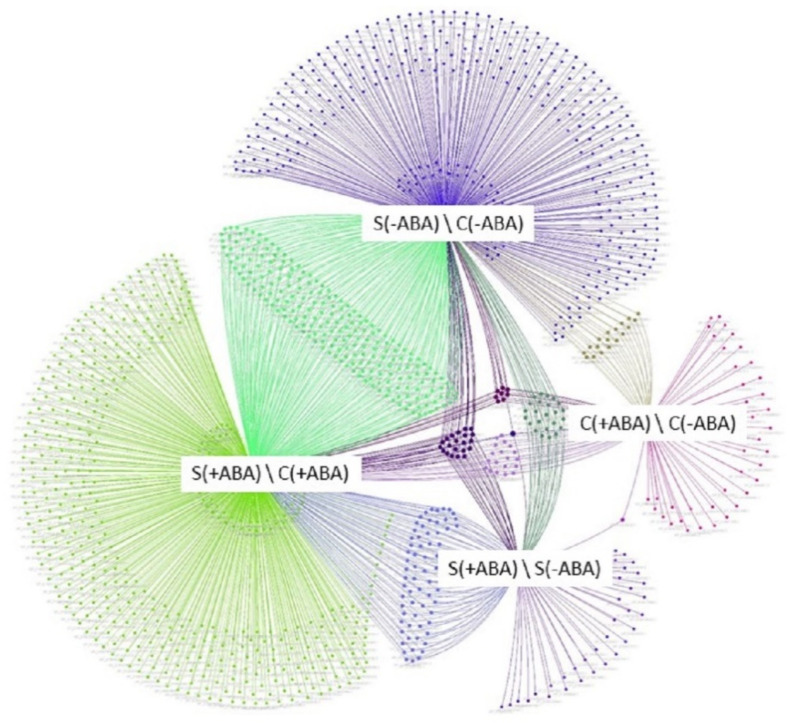
Venn network analysis [25] of differentially expressed proteins (DEPs) identified in the four experimental comparisons. The overlapping/connected edges indicate the DEPS shared between corresponding groups. The green, purple, red and dark blue colour node areas represent DEPs from S(+ABA)\C(+ABA), S(−ABA)\C(−ABA), C(+ABA)\C(−ABA) and S(+ABA)\S(−ABA), respectively.

Pairwise comparisons of before and after stress exposure, and with and without ABA pre-treatment, were used to identify differentially expressed proteins (DEPs), defined as those proteins with a fold-change of >1.5 or <0.67, and *p*-value < 0.05. With reference to Figure 2, the combinations of the four comparisons reflect the impact of multiple abiotic stress or ABA pre-treatment on IAC1131 rice plants. A comparative study on the multiple abiotic stress proteome was performed between the ABA incubated and non-incubated stressed conditions [S(+ABA), S(−ABA)] and control conditions [C(+ABA), C(−ABA)], giving four comparison groups.

A total of 87 differentially abundant proteins were identified between ABA incubated and ABA non-incubated control plants [C(+ABA)\C(−ABA)]. Of these DEPs, 38 were at higher abundance levels in the ABA incubated compared to ABA non-incubated group, while 49 were reduced in abundance. After multiple abiotic stress treatment, we found 148 DEPs between ABA incubated and ABA non-incubated plants [S(+ABA)\S(−ABA)]. Of these DEPs, 85 had higher abundance levels in the ABA incubated group in comparison with ABA non-incubated plants, while 63 were reduced in abundance.

**Figure 3:**
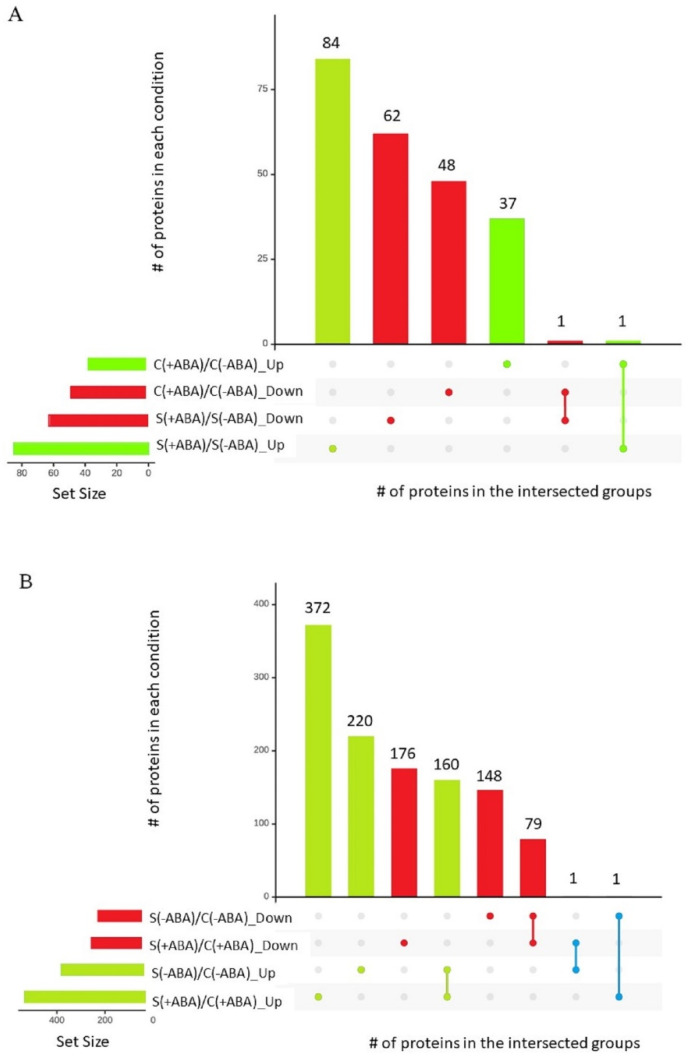
Upset plot of the number of proteins that increased and decreased in abundance significantly between two different comparisons. (**A**) Proteins expressed significantly in comparisons of ABA incubated and ABA non-incubated conditions. (**B**) Proteins expressed significantly in comparisons of stress and control conditions.

Interestingly, peptide chain release factor PrfB1 (XP_015647593.1) was the only protein that increased in abundance under both the control and stress conditions between ABA incubated and non-incubated plants, while the Leucine-rich repeat (LRR) family protein (XP_015633612.1) was the only protein that decreased in abundance under both the control and stress conditions between ABA incubated and non-incubated plants (Figure 3A).

### 2.4. Effect of Multiple Abiotic Stress on Rice Plants with and without ABA Pre-Treatment

Pairwise comparisons were performed to quantify the number of DEPs between stressed and control samples, incubated and non-incubated with ABA. The ABA pre-treated samples resulted in 789 DEPs under multiple abiotic stress compared with the control condition [S(+ABA)\C(+ABA)]. Of these, more than 60% (533) increased in abundance. Analysis of IAC1131 plants without ABA pre-treatment identified 609 proteins differentially expressed in response to multiple abiotic stress. Of these, more than 60% (381 proteins) responded positively to multiple abiotic stress treatments compared with the control plants [S(−ABA)\C(−ABA)]. Notably, two proteins showed contrasting changes in abundance between these two comparisons. Peptidyl-prolyl cis-trans isomerase FKBP18 (XP_015627276.1) increased in abundance in response to stress with ABA pre-treatment but decreased in response to stress in the absence of ABA pre-treatment, while oryzain beta chain protein (XP_015636103.1) reduced in abundance in response to stress with ABA pre-treatment but increased in response to stress in the absence of ABA pre-treatment (Figure 3B). In total, there were 1633 DEPs among the four comparison groups, as shown in Table 1. Details of all proteins and DEPs identified in all four comparisons are provided in Supplementary Data File S1.

### 2.5. Identification of Differentially Expressed Proteins Modulated by ABA in Control Conditions

**Figure 4:**
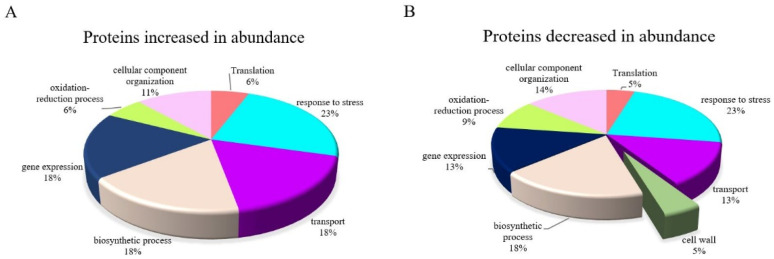
The effect of leaf-sprayed ABA on biological functions of IAC1131 plants grown under control conditions. (**A**) Seven major biological processes trended positively by increasing the abundance of related proteins significantly. (**B**) Eight major biological processes trended negatively by decreasing the abundance of related proteins significantly.

Interrogating the DEPs between the two sets of plants grown under control conditions, one group of which was pre-treated with ABA and one group of which was not, would illustrate the effect of exogenous ABA on normal IAC1131 plants. The major biological functions affected by ABA both positively and negatively in these plants are presented in Figure 4. Seven functions responded to ABA treatment by increasing the abundance of related proteins, including translation, response to stress, transport, biosynthetic process, gene expression, an oxidation-reduction process and cellular component organization (Figure 4A). Proteins related to similar biological functions decreased in response to ABA treatment, accompanied by a reduction in cell wall proteins. Among these, a cell-wall localized functional protein, alpha-galactosidase isoform X1 protein (XP_015613566.1), was the most significantly decreased with a −5.37-fold change (Figure 4B).

### 2.6. Identification of Differentially Expressed Proteins Modulated by ABA in Stress Conditions

**Figure 5:**
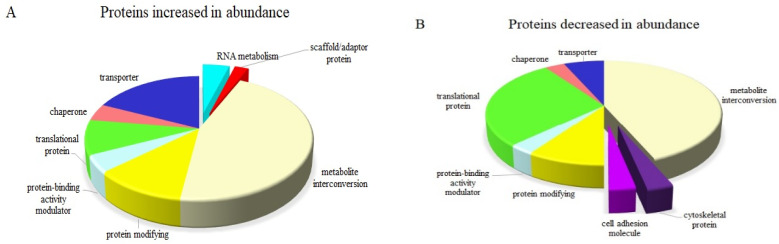
Differentially expressed proteins modulated by ABA in stressed IAC1131 plants compared with non-ABA-incubated plants. (**A**) Eight major protein groups trended positively by increasing the abundance of related proteins significantly. (**B**) Eight major protein groups trended negatively by decreasing the abundance of related proteins significantly.

A total of 148 differentially expressed proteins were identified between the stressed plants incubated with ABA and stressed plants non-incubated with ABA, which included 85 and 63 proteins that increased and decreased in abundance, respectively. Functional annotation analysis revealed that GO-enriched identified proteins fall into different functional protein groups with obvious differences. RNA metabolism and scaffold/adaptor proteins exclusively increased with ABA pre-treatment in stressed plants, while cell adhesion and cytoskeleton-related proteins exclusively decreased (Figure 5). Interestingly, ABA stimulated both translational and transport proteins by decreasing and increasing in abundance; however, the number of transporter proteins that increased in abundance was three times greater than those that decreased in abundance, while a converse difference was observed for translational-related proteins.

### 2.7. Stress-Responsive Proteins Regulated by ABA Incubation

**Figure 6:**
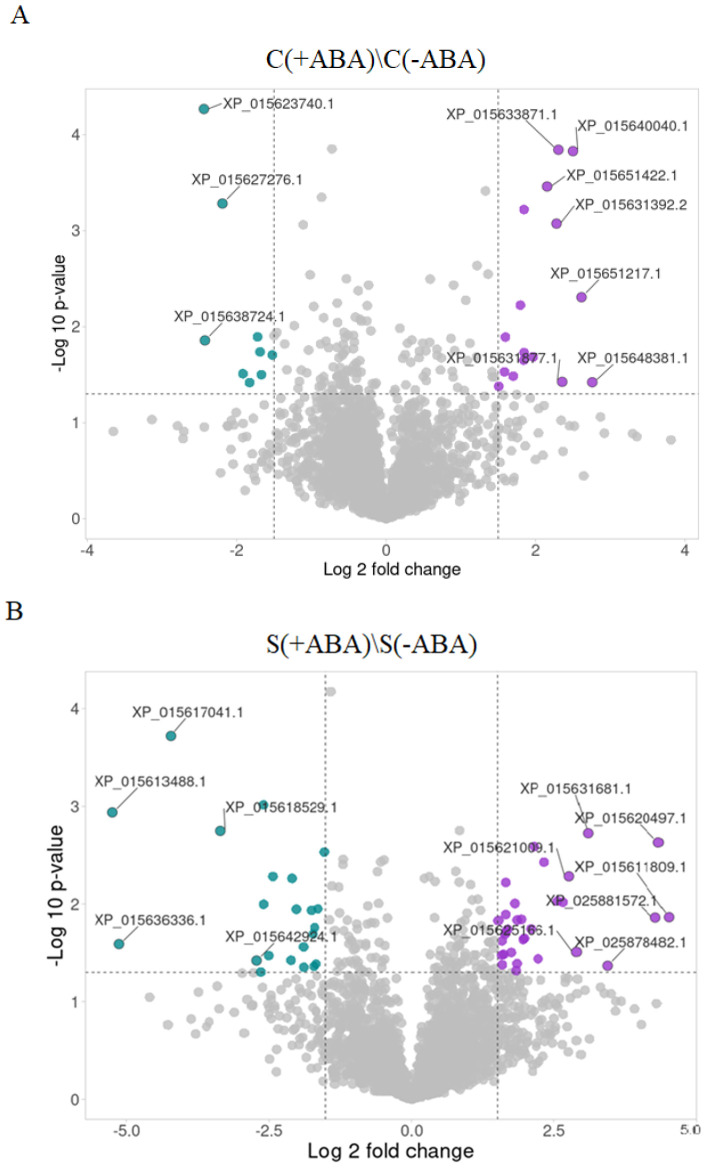
Differentially expressed proteins modulated by ABA in IAC1131 plants compared with non-ABA-incubated plants in both the control and stressed groups. (**A**) Volcano plot for proteins and DEPs identified in ABA-treated control rice samples compared with untreated plants. (**B**) Volcano plot for proteins and DEPs identified in ABA pre-treated plants compared with no ABA pre-treatment, after exposure to multiple abiotic stress. Each point represents a protein with log 2-fold change along the *x*-axis and −log10, *p*-value along the *y*-axis. Purple, green and grey points show the increased, decreased and unchanged proteins, respectively. Dashed lines show the *p*-value of 0.05 cut-off along the *y*-axis, and FC > 1.5 cut-off for proteins that increased in abundance and FC < 1.5 cut-off for proteins that decreased in abundance along the *x*-axis.

ABA pre-treatment increased the abundance of a significant number of proteins in both the control and stressed IAC1131 plants, as shown in Figure 6. An observation of the proteome response amplitude showed a greater change in the expression level (fold change value) for the stressed plants and a clear distinction between the control and stressed plants. The fold change extreme for the control group comparison [C(+ABA)\C(−ABA)] was between 6.8 and −5.4, while for the stressed plants comparison [S(+ABA)\S(−ABA)], the fold-change values ranged between 22.9 and −37.9.

The consequence of ABA application on the control plant comparison [C(+ABA)\C(−ABA)] was that 38 proteins increased in abundance and 49 proteins decreased in abundance. The proteins that increased the most by ABA application included nuclear transcription factor Y subunit C-6 (XP_015648381.1, FC = 6.77), protein detoxification 16 (XP_015651217.1, FC = 6.13), ADP, ATP carrier protein 1 (XP_015640040.1, FC = 5.66), mitochondrial ATP synthase 6 kDa subunit (XP_015631877.1, FC = 5.12) and protein-L-isoaspartate O-methyltransferase (XP_015633871.1, FC = 4.95). On the other hand, proteins with the greatest decrease in abundance included low PSII accumulation 2 (XP_015623740.1, FC = −5.42), outer envelope pore protein 16 (XP_015638724.1, FC = −5.37) and peptidyl-prolyl cis-trans isomerase FKBP18 (XP_015627276.1, FC = −4.57) (Figure 6A).

The proportion of proteins that increased in abundance in response to ABA application after exposure to multiple abiotic stress was about 15% greater than the number of proteins reduced in abundance. Proteins with the greatest change in abundance, with a fold change between 8.5 and 22.9, included phosphatase 2C 70 isoform X2 (XP_015611809.1, FC = 22.86), proline-tRNA ligase (XP_015620497.1, FC = 20.14), mitogen-activated protein kinase 9-like (XP_025881572.1, FC = 19.45), BRI1-KD interacting protein 135 (XP_025878482.1, FC = 10.81) and TATA-binding protein 2 isoform X1 (XP_015631681.1, FC = 8.53). The fact that all these proteins greatly increased in abundance in the presence of exogenous ABA (Figure 6B) suggests they are strongly influenced by ABA signalling and hence may play important roles in mediating plant multiple abiotic stress responses.

To investigate the effect of ABA treatment on IAC1131 plant response to multiple abiotic stress, we examined the expression profile of proteins that decreased in abundance in comparison between stressed plants with and without ABA pre-treatment. Late embryogenesis abundant protein 14-like protein (XP_015613488.1) declined the most (FC = −37.94), and four other proteins with the largest decreases in abundance included 2,3-dimethylmalate lyase-like protein (XP_015636336.1, FC = −34.98), TOM1-like protein 3 (XP_015617041.1, FC = −18.73), GEM-like protein 5 (XP_015618529.1, FC = −10.18) and splicing factor 1 (XP_015642924.1, FC = −6.60). Further study of the biological processes and molecular functions of these proteins would deepen our understanding of the regulatory collaboration between ABA signalling and stress response (Figure 6B).

In order to elucidate the proteins involved in governing the ABA signalling response in IAC1131 leaves irrespective of stress, a comparison between the results of the two conditions [C(+ABA)\C(−ABA)] and [S(+ABA)\S(−ABA)] was performed. ABA induced changes in abundance of only two proteins under both the control and stressed group plants when compared with no ABA treatment (Figure 4). Peptide chain release factor PrfB1 (XP_015647593.1) was the only protein that accumulated positively in both the control and stressed plants when incubated with ABA. Conversely, the Leucine-rich repeat (LRR) family protein (XP_015633612.1) exhibited significantly lower accumulation in both groups of plants pre-treated with exogenous ABA. These results indicate that the expression of the peptide chain release factor PrfB1 protein is dependent on ABA, and thus it would be interesting to investigate if it plays a significant role in ABA signalling.

### 2.8. Multiple Abiotic Stress-Responsive Proteins Correlated with ABA Pre-Treatment

A total of 609 non-redundant proteins were significantly altered in abundance in IAC1131 plants in comparisons between multiple abiotic stress and control conditions, in the absence of ABA pre-treatment. Of these proteins, 381 increased in abundance and 228 proteins decreased in abundance, as shown in the Upset plot [26] in Figure 3. These DEPs can be considered as ABA-independent proteins involved in multiple abiotic stress response. When plants were pre-treated with ABA, an additional 180 proteins were differentially accumulated in response to stress. Most of this difference (152 proteins) were DEPs that increased in abundance. This increased number of stress-responsive proteins, most of which increased in abundance, could be indicative of the positive effect of ABA pre-treatment on plant response to multiple abiotic stress.

Generally speaking, ABA pre-treatment on multiple abiotic stress-exposed plants induced different protein expression trends when compared to the control plants. ABA pre-treatment of plants exposed to multiple abiotic stress induced significant changes in abundance of a greater number of proteins, with 148 DEPs compared to 87 DEPs in plants grown in control conditions, with ABA pre-treatment (Figure 3). Additionally, DEPs in stressed plants pre-treated with ABA mostly increased in abundance (85 out of 148), while a majority of the DEPs in plants grown under control conditions and pre-treated with ABA decreased in abundance (49 out of 87) (Figure 3B).

The tricarboxylic acid (TCA) cycle, also known as the Krebs cycle, is one of the vital energy pathways and an important part of respiration mechanisms in living systems. The TCA cycle is a series of eight enzymes primarily linking the oxidation of pyruvate and malate to CO_2_ for oxidation by the mitochondrial respiratory chain [27]. We analysed the correlation of proteins (enzymes) in the TCA cycle which increased in abundance with ABA signalling or stress response in plants. Two comparisons were examined in order to investigate the regulation of proteins of the TCA cycle under ABA pre-treatment and multiple abiotic stress treatment: [S(−ABA)\C(−ABA)] and [S(+ABA)\C(+ABA)].

**Figure 7:**
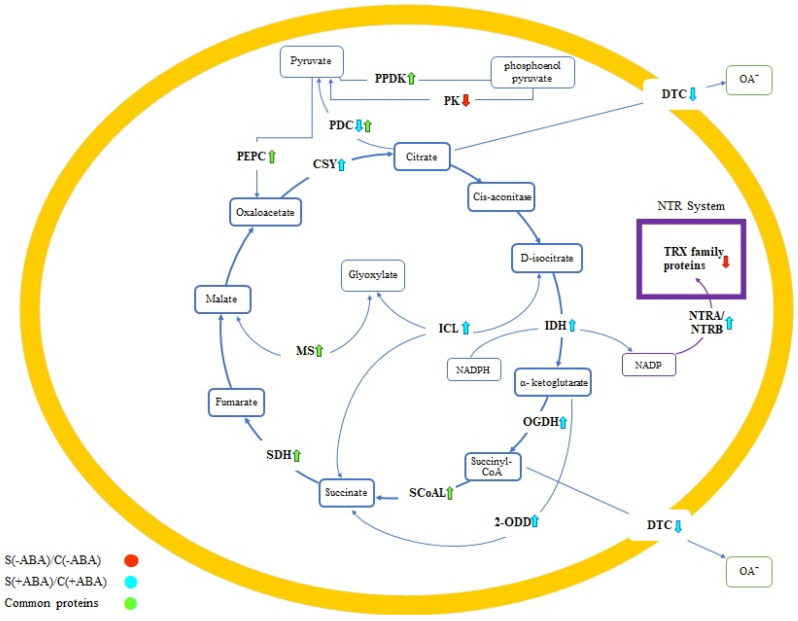
A schematic summary of the expression of enzymes related to the TCA cycle in plant cell mitochondria in IAC1131 plants under multiple abiotic stress, with and without ABA application. Differentially abundant proteins under [S(−ABA)\C(−ABA)] are highlighted in red, [S(+ABA)\C(+ABA)] are highlighted in blue and shared proteins between the two comparisons are highlighted in green. Upward and downward arrows indicate proteins that increased and decreased in abundance, respectively. CSY: citrate synthase; ICL: isocitrate lyase; IDH: isocitrate dehydrogenase; OGDH: 2-Oxoglutarate dehydrogenase; 2-ODDs: 2-Oxoglutarate dependent dioxygenases; SCoAL: succinyl-CoA synthetase; SDH: succinate dehydrogenase; MS: malate synthase; PK: pyruvate kinase; 2OG: 2-oxoglutarate; PPDK: pyruvate phosphate dikinase; PDC: pyruvate dehydrogenase complex; PEPC: phosphoenolpyruvate carboxylase; DTC: dicarboxylate/tricarboxylate carrier; TRX: thioredoxins; NTR: NADPH-Trx system; NTRA/NTRB: NADPH-Trx reductase A/B.

Five out of the eight TCA cycle primary enzymes exhibited significant increases in abundance in at least one of these comparisons, including isocitrate dehydrogenase (IDH), 2-oxoglutarate dehydrogenase (OGDH), succinyl-CoA synthetase (SCoAL), succinate dehydrogenase (SDH) and citrate synthase (CSY). Among these, SCoAL and SDH were accumulated in response to both multiple abiotic stress and exogenous ABA application, while IDH, OGDH and CSY increased in abundance in response to multiple abiotic stress with ABA pre-treatment (Figure 7). The latter three enzymes could thus be considered as both ABA signalling and stress-responsive proteins.

To further confirm the effect of ABA on carbon metabolism, we analysed the expression of enzymes related to the TCA cycle in plants. The TCA cycle intersects with several metabolic pathways in mitochondria by adding the cycle intermediates, and many of these enzymes were changed in abundance in IAC1131 plants under ABA incubation when compared to normal treatments. For instance, pyruvate phosphate dikinase (PPDK), phosphoenolpyruvate carboxylase (PEPC) and malate synthase (MS) increased in abundance in all plants after exposure to multiple abiotic stress, while different isoforms of the pyruvate dehydrogenase complex (PDC) either increased in both comparisons or decreased in the [S(+ABA)\C(+ABA)] comparison. The other negative effect of ABA application on plant response to stress conditions was evident in dicarboxylate/tricarboxylate carrier (DTC) enzymes, where protein abundance decreased exclusively in ABA pre-treated plants. Our results show that the above enzymes are ABA-dependent and play a role in ABA signalling in plants, such that addition of exogenous ABA (which increases ABA concentration) leads to an increase in their accumulation when plants are facing multiple abiotic stress.

## 3. Discussion

### 3.1. Does ABA Application Help Plants in Normal Conditions?

One approach to enhance plants’ tolerance to environmental imbalance has been to overproduce ABA [28]. Plants identify ABA as a stress hormone; therefore, they respond to ABA signalling and balance growth programs against stress responses [15,22]. However, evidence has demonstrated that induction of ABA over-accumulation in plants, such as by high concentrations of exogenously applied ABA, can result in growth arrest [29,30]. Hence, ABA over-accumulation under non-stress conditions could be sensed by plants as a stress condition, leading to a reduction in growth [31]. However, the mode of ABA-mediated growth regulation varies depending on ABA concentration, timing and tissues, showing both positive and negative effects on growth, depending on the specific context and circumstances [32,33].

Biological function analysis of the control IAC1131 plants pre-treated, or not, with ABA revealed the cell-wall function as the only function responding negatively to exogenous ABA application. One of the main proteins related to this function, alpha-galactosidase, was one of the DEPs which most significantly decreased in abundance. Galactosidase family proteins are cell-wall modifying enzymes which play roles in numerous different plant biological functions [32,34]. These enzymes have been recently shown to be hormone-responsive elements that increase in abundance after treatment with ethylene [35], a plant hormone that plays an important role in leaf senescence, in contrast with ABA which is responsible for enhancing leaf growth. ABA over-accumulation limits ethylene biosynthesis in plants and enhances leaf tissue conductance and water transfer in plants [36]. Likewise, our finding that exogenous ABA negatively affected alpha-galactosidase may be linked indirectly to decreasing ethylene concentration in plants, resulting in a cell-wall function decline and enhancing water transfer to leaf tissues.

### 3.2. Does Exogenous ABA Application Help Plants in Stress Conditions?

ABA is an important phytohormone which interacts with the signalling molecules of processes involved in stress response; hence, it is commonly known as the ‘stress hormone’ [37]. The mechanisms by which plants respond to stress, however, include both ABA-dependent and ABA-independent processes [38].

Our research demonstrated that ABA pre-treatment altered the proteome profile of IAC1131 plants in response to multiple abiotic stress by inducing the expression of 148 proteins during stress. Approximately 57% of these proteins responded positively to exogenous ABA application and increased in abundance. This could be a signal of an ABA effect on stress mitigation, whereby exogenous ABA supports endogenous ABA functions in inducing plant tolerance to cope with environmental stress. It has been suggested previously that overloaded ABA signalling, including exogenous ABA in addition to endogenous ABA accumulation, can reduce negative effects of stress on plants [39].

An analysis of biological functions under stress conditions, with and without ABA incubation, is important to gain a better understanding of the commonality of ABA signalling proteins with stress-responsive proteins, and crosstalk between them. Metabolite interconversion was the most responsive function to stress, followed by alterations in protein modification, chaperone, protein-binding activity modulator, transporter and translation functions. These functions could be considered as defences activated against stress injury after ABA over-accumulation.

Notably, ABA incubation resulted in transport and translation functions changing in an opposite manner in response to stress; transport increased, while translation decreased. ABA accumulation affects the membrane ion transport systems during osmotic stress, not only as a communicator between roots and shoot but also by efficiently controlling the water status in plants under unfavourable conditions [40]. Some translational- and post-translational-related proteins have been defined as ABA-dependent stress-responsive proteins [41], but the underlying molecular mechanisms, including those responsible for the greater number of proteins that decreased in abundance after ABA treatment and multiple abiotic stress, are still unclear and deserving of further investigation.

Transcription factors accumulate under environmental stresses due to the overexpression of stress-responsive genes. Some of them, such as transcription factor ABF2, are ABA-dependent factors; therefore, both ABA over-accumulation and stress conditions can be positive regulators of RNA metabolism [42]. ABA pre-treatment on the IAC1131 plants exposed to multiple abiotic stress enhanced scaffold protein functions, including RNA metabolism.

Ubiquitin proteins are known to regulate protein function by post-translational modifications, and Cullin RING ligase enzymes (CRLs) mediate the ubiquitination process under stress conditions. ABA is a positive regulator of CRLs, similar to certain specific transcription factors, and plays a central role in response to abiotic stresses [43]. This suggests that ABA pre-treatment in the IAC1131 rice plants is associated with a positive regulation of RNA metabolism and post-translational functions and a negative regulation of cell-wall functions.

### 3.3. Is the TCA Cycle a Stress Response or an ABA Signalling Cycle?

Previous studies indicated the effect of exogenous ABA treatment on inducing environmental stress resistance in plants. On the other hand, an increase in ABA levels after exposure to stress has also been reported [44]. Therefore, a characterization of the ABA over-accumulation before and after stress conditions might identify the mutual effect of ABA on abiotic stress response and abiotic stress pressure on ABA signalling in plants.

Finding the sites of ABA action is inherently important in understanding the signalling network of ABA. Various studies have documented that the ABA response to external stresses is a highly complex network mechanism. Knowledge of stress signals and responses, with and without the addition of exogenous ABA, can help to elucidate the role of ABA at the molecular level in response to multiple abiotic stress and provide new insights for future studies.

Although ABA-dependent and ABA-independent signalling pathways function cooperatively to enhance stress tolerance in plants [45], our findings showed that ABA pre-treatment helped IAC1131 plants to respond to multiple abiotic stress, with a greater number of DEPS that increased in abundance in comparison with plants exposed to the same stress conditions without ABA pre-treatment. Increasing the number of stress-responsive proteins by about 65% after incubation with ABA, with a 70% increase in abundance, is indicative of the positive regulatory effect of ABA levels on environmental stress response in rice plants.

The tricarboxylic acid (TCA) cycle is one of the canonical energy sources of living systems, and in plants, it is composed of a set of eight dynamic enzymes primarily linking the oxidation of pyruvate and malate (generated in the cytosol) to CO_2_ for oxidation, as an important part of aerobic respiration [27], as well as to a wide range of regulatory mechanisms such as phosphorylation, light-dependent regulation and acetylation [46]. Previous studies have illustrated the connection between the TCA cycle and ABA accumulation in Arabidopsis plants under unstressed conditions [47] and highlighted the role of the TCA cycle in the response of maize plants to salt stress [48].

The ABA treated plants grown under stress conditions, [S(+ABA)\C(+ABA)], showed an increased accumulation of two important enzymes in the TCA cycle, succinate dehydrogenase (SDH) and succinyl-CoA synthetase (SCoAL). This was similar to the stress response in plants that were not pre-treated with ABA, [S(−ABA)\C(−ABA)]. Three out of the eight main TCA cycle enzymes exclusively responded positively in ABA pre-treated samples exposed to stress conditions, namely, isocitrate dehydrogenase (IDH), 2-oxoglutarate dehydrogenase (OGDH) and citrate synthase (CSY). Interestingly, OGDH was detected as one of the proteins that increased in abundance when the control plants treated with ABA were compared with the untreated plants [C(+ABA)\C(−ABA)]. In agreement with this outcome, an increase in OGDH was observed in the ABA-biosynthesis mutant Arabidopsis plants [47].

Dicarboxylate/tricarboxylate carrier (DTC) and pyruvate dehydrogenase complex (PDC) were two enzymes related to the TCA cycle that decreased in abundance in the ABA pre-treated plants subjected to stress [S(+ABA)\C(+ABA)]. One of the purposes of mitochondrial oxidation of malate is to produce citrate in the TCA cycle, thus inducing inactivation of pyruvate dehydrogenase and allowing pyruvate to feed into citrate [49]. However, recent findings have revealed the positive regulation of DTC enzymes under stress, which supports the function of this enzyme in reduction in citrate export from the TCA cycle [50,51]. This might reflect the negative consequence of ABA over-accumulation on plant response to stress.

Pyruvate kinase (PK) and thioredoxins (TRX) responded negatively to multiple abiotic stress conditions in ABA untreated plants [S(−ABA)\C(−ABA)]. Pyruvate kinases play an important role in coordinating other metabolic pathways, and the reduction of TCA activity under mitochondrial stress, with downregulation of PK, might limit the flow of intermediates into the TCA cycle [52].

The NTR system of thioredoxin reductases is correlated with TCA function, with some of these enzymes linked to the TCA cycle. NADPH-Trx reductase A/B (NTRA/TRB) was detected in our results, with an increased abundance in stressed plants in comparison with the control when pre-treated with ABA, which highlights the effect of TCA cycle regulation on the activity of this enzyme under stress. Interestingly, a member of the TRX protein family (a thioredoxin-like protein) decreased similarly in response to stress treatment but not ABA treatment. Due to the inverse correlation between succinate dehydrogenase (SDH) enzyme activity and the accumulation of NTR complex enzymes, an increase in SDH activity is known to result in a decline in TRX proteins [53].

### 3.4. Do Biological Analysis and Mathematical Analysis Produce the Same Results?

Four comparisons between the IAC1131 plants under different conditions were used to investigate proteome function under stress, in the presence or absence of exogenous ABA application. In this context, it would be interesting to compare a mathematical analysis with biological findings.

Considering it as a simple mathematics problem: the sum of the DEPs resulting from the comparisons of [C(+ABA)\C(−ABA)] and [S(+ABA)\S(−ABA)], which represents the sum of the ABA response in both the stressed and non-stressed conditions, should be approximately equal to the number of DEPs found in [S(+ABA)\C(+ABA)] minus [S(−ABA)\C(−ABA)], which represents the difference between the stress response in the presence of ABA and the stress response in the absence of ABA. Both sides of this equation should be equal to the DEPs involved in the ABA response. The sum of 87 and 148 DEPs (from C(+ABA)\C(−ABA)] and [S(+ABA)\S(−ABA)]) is 235 DEPs. The difference between the 789 DEPs in [S(+ABA)\C(+ABA)] and the 609 DEPs in [S(−ABA)\C(−ABA)] is 180 DEPs. These values are within 30% of each other, which is surprisingly close and indicates that even though plant biology involves the analysis of dynamic and unpredictable living organisms, the mechanisms of operation are underpinned by mathematically precise biological system engineering!

## 4. Materials and Methods

### 4.1. Plant Material and Stress Treatment

Prior to sowing, seeds were sterilized in four steps: 70% ethanol for 20 min, water washing for 1 min, 50% bleach solution for 30 min and final water washing for 5 min. Five seeds of (*Oryza sativa*) rice genotype IAC1131 were sown in the same pots (30 cm deep and 10 cm in diameter) filled with 700 g for each pot, with the soil mix consisting of 25% (*v*/*v*) Waikerie river sand, 25% (*v*/*v*) peatmoss, 35% (*v*/*v*) peat mix and 15% (*v*/*v*) clay loam, supplemented with NPK 23:4:14 fertilizer. There were 12 pots in total, comprising three biological replications for all four treatment regimens applied. Plants were fertilized twice, the first time simultaneously with soil filling, and again after 2 weeks. The plants were grown in a greenhouse under controlled conditions with the temperature set to 28/22 °C (day/night), a 12 h photoperiod and with the light intensity set to a minimum of 700 µmol m^−2^ s^−1^.

Plants of the IAC1131 genotype of rice were grown to the vegetative stage and, after four weeks of growth, plants were divided into four groups, each one comprising three plants. The first group was sprayed with distilled water and kept under well-watered conditions, in which plants were watered every day up to full field capacity (100%) and labelled as (Control − ABA) plants. The second group was sprayed with 100 μM ABA hormone for 2 days consistently and kept under well-watered conditions, in which the plants were watered every day up to full field capacity and labelled as (Control +ABA) plants. The third group was sprayed with distilled water and kept under well-watered conditions prior to the imposition of multiple abiotic stress treatment for 4 days and labelled as (Stress − ABA). The last group of plants was sprayed with 100 μM ABA hormone for 2 days consistently prior to the imposition of multiple abiotic stress treatment for 4 days and labelled as (Stress + ABA). 

After a period of 2-day ABA or distilled water spraying and 2-day stress pre-treatment, multiple abiotic stress was applied, which consisted of 50% field capacity as drought stress, 50 mM NaCl as salt stress and 30/18 °C as temperature stress. To achieve simultaneous drought and salt stress, 2 days before starting the combined stress treatment, plants were watered with 25 mM NaCl to reach 100% field capacity with 25 mM NaCl concentration, then watering was stopped till the field capacity reduced to 50%. The amount of water transpired by plants was recorded daily by weighting the pots, and multiple abiotic stress treatment was started by applying temperature stress at 30/18 °C (day/night). After 0 days (control) and 4 days (stress), fresh leaves were used for measuring physiological parameters, and additional leaves were collected in three replications and lyophilized immediately in liquid nitrogen. For further experiments, samples were placed in 2 mL centrifuge tubes and ground finely using a Qiagen Retsch 12090 TissueLyser II instrument, five Zironox beads (2.8–3.3 mm) and liquid nitrogen.

### 4.2. Protein Extraction and Assay

Protein extraction was performed as described previously [54,55], with 50 mg of leaf powder initially suspended in 1.5 mL of 10% trichloroacetic acid in acetone, 0.07% β-mercaptoethanol and incubated at −20 °C for 45 min. The extract was centrifuged for 15 min at 16,000× *g* at 4 °C, and the pellet was collected and washed with 1.5 mL of 100% acetone followed by centrifugation for 15 min at 16,000× *g* at 4 °C. The acetone washing step was repeated three times for the complete removal of pigments, lipids and other lipophilic molecules. The resulting colourless pellet was lyophilized in a vacuum centrifuge for 5 min. A total of 400 µL of 2–3% SDS in 50 mM Tris-HCl (pH 8.8) was used to resuspend the pellet. After shaking for 2 h, the pellet was removed, and the samples were reduced by adding 1 M Dithiothreitol to reach a final concentration of 10 mM and then incubated for 1 h at 37 °C followed by alkylation with 20 mM Iodoacetamide for 45 min in the dark at room temperature.

Samples were then methanol-chloroform precipitated. A total of 300 µL of protein solution was mixed with 800 µL of methanol and 200 µL of chloroform. A total of 500 µL of water was added to the mixture, vortexed and centrifuged at 6000× *g* for 2 min. After removing the upper phase, 600 µL of methanol was added to the mixture and centrifuged at 6000× *g* for 2 min, and the supernatant was removed. The pellet was air-dried and solubilized in 80 µL of 8 M urea in 100 mM Tris-HCl (pH 8.8) buffer. The concentration of protein in the solution was measured by a bicinchoninic acid (BCA) assay kit (Pierce, Thermo Fisher Scientific, San Jose, CA, USA).

### 4.3. Trypsin In-Solution Digestion and Peptide Extraction

A total of 100 µg aliquots of protein were used for digestion and peptide extraction. Samples were diluted five times with 100 mM Tris-HCl buffer (pH 8.8) to reduce the concentration of urea to less than 2 M. For peptide digestion, 1:50 trypsin enzyme to protein was added and incubated overnight at 37 °C. The reaction was stopped by adding trifluoroacetic acid (TFA) to reach a final concentration of 1%. After acidifying samples with TFA, they were desalted by using an SDB-RPS stage-tip (3 M, Saint Paul, MN, USA). Samples were spun and loaded in stage tips (4 punches of SDB membrane in 200 µL pipette tips), held by adaptors in 2 mL tubes and centrifuged at 2500 rpm [56]. The tips were washed twice with 200 µL of 0.2% TFA, then 200 µL each of 80% acetonitrile (ACN) and 5% NH_4_OH were added and centrifuged at 2500 rpm. Eluted peptides were evaporated to dryness in a vacuum centrifuge then reconstituted in 0.1% formic acid. Peptide concentration was measured using a micro-BCA kit (Pierce, Thermo Fisher Scientific, San Jose, CA, USA).

### 4.4. High pH Reversed-Phase Fractionation of Peptides

An aliquot of all 12 digested samples (consisting of three replicates, each from four conditions) was pooled and fractionated using a High pH Reversed-Phase Peptide Fractionation Kit (Pierce, Thermo Fisher Scientific). Briefly, 40 µg of the mixed peptides from the pooled sample was diluted with 300 μL of 0.1% TFA. Eight gradient fractions of ACN (5%, 7.5%, 10%, 12.5%, 15%, 17.5%, 20% and 50%) were collected using the appropriate elution solutions, in addition to the flow-through and wash fractions. Finally, the samples (10 fractions) were evaporated to dryness in a vacuum centrifuge and reconstituted with 20 µL of 0.1% formic acid.

### 4.5. Data-Dependent Acquisition Shotgun Proteomic Analysis

Data-dependent and data-independent acquisitions (DDA and DIA, respectively) were performed as described previously [24,57]. Nanoflow LC-MS/MS was performed using a Triple TOF 6600 mass spectrometer (Sciex, Framingham, MA, USA) equipped with an Eksigent nanoLC 400 liquid chromatography system (Sciex) and nanoflex cHiPLC module (Sciex). HpH fractionated peptides were injected onto a reversed-phase trap (Halo-C18, 160 Å, 2.7 µm, 150 µm × 3.5 cm) for pre-concentration and desalted with a loading buffer. The peptide trap was then switched in line with the analytical column (Halo-C18, 160 Å, 2.7 µm, 200 µm × 20 cm). Peptides were eluted from the column using a linear solvent gradient of 5–35% of mobile phase B (0.1% formic acid, 99.9% acetonitrile) over 60 min at a flow rate of 600 nL/min [58]. The reversed phase nano-LC eluent was subject to positive ion nanoflow electrospray analysis in DDA mode. A TOF-MS survey scan was acquired (*m*/*z* 350–1500, 0.25 s), with the 20 most intense multiply charged ions (2+ to 4+; exceeding 200 counts/s) in the survey scan being sequentially subjected to MS/MS analysis. MS/MS spectra were accumulated for 100 milliseconds in the mass range *m*/*z* 100–1800 using rolling collision energy.

### 4.6. Data Independent Acquisition by Sequential Windowed Acquisition of All Theoretical Mass Spectra

For SWATH-MS, peptides of the 12 individual sample replicates were separated over an RP linear gradient using the same LC and MS instruments as specified above, with positive ion nanoflow electrospray mode. In SWATH mode, first a TOF-MS survey scan was acquired (*m*/*z* 350–1500, 0.05 s), then 100 predefined *m*/*z* ranges were sequentially subjected to MS/MS analysis. A total of 100 variable windows were selected based on the intensity distribution of precursor *m*/*z* in the previously acquired DDA data. MS/MS spectra accumulated for 40 milliseconds in the mass range *m*/*z* 350–1500 with rolling optimized collision energy. To minimize sample carryover, blank injections were performed between every sample injection. Additionally, sample data were acquired in a randomized order to avoid batch effect biases.

### 4.7. Peptide to Spectrum Matching and Data Processing

Data generated in the DDA mode were processed using the Paragon algorithm in ProteinPilot ver 5.0 (Sciex) using default parameters and the NCBI *O. sativa* protein sequence database (March 2022, 42,578 sequences). ProteinPilot DDA search results were imported into PeakView ver 2.2 (Sciex) and used to create a spectral library. To align retention times for all SWATH files, a linear regression was applied for each sample type by using at least five manually selected peptides across the elution profile. The following search parameters were used for quantitation: top six most intense fragment ions for each peptide, a maximum number of peptides of 100, 75 ppm mass tolerance, 99% peptide confidence threshold, 1% FDR threshold and a 5 min retention time extraction window.

The peak areas for peptides were extracted by summing the areas under curve values of the corresponding fragment ions using PeakView. The summed peak areas of the peptides were normalized against the total abundance values of respective samples, then used for protein quantification. To assess differentially expressed proteins (DEPs), a comparison of protein abundances across respective sample groups was performed using two-sample Student’s *t*-tests. Proteins with *p* < 0.05 and an expression fold-change of >1.5 or less than 0.67 were considered significantly changed between treatments [59].

### 4.8. Bioinformatics Analysis of Proteomic Data

Web-based MetaboAnalyst ver 5.0 software [60] was applied for Pearson correlation and partial least square discrimination analysis (PLS-DA) for all samples. PLS-DA was performed to find the greatest contributions of variables to the differences between samples based on the variable of importance in projection score values (VIPs) [61].

## 5. Conclusions

Our findings shed light on how exogenous application of the ABA hormone relates to stress-responsive metabolism in rice plants. Physiological parameters reveal no statistically significant effects of ABA application on the IAC1131 plants grown under control conditions, and likewise, the proteomics data illustrate that most of the same biological functions are expressed in plants grown under control conditions, whether they are pre-treated with ABA or not. However, ABA application on plants subjected to multiple abiotic stress greatly increased the number of DEPs across a range of biological functions Therefore, ABA pre-treatment acts synergistically with stress-response proteins in plants.

The TCA cycle is known as a stress regulator in plants, and use of ABA as a pre-treatment highlighted the positive role of ABA in enhancing the TCA cycle activity under unfavourable conditions, by increasing the abundance of the main active enzymes. Our results support the hypothesis that ABA signalling proteins play complex roles in the context of abiotic stress response in plants and suggest a mutual relationship between ABA over-accumulation and stress tolerance. The increase in biosynthesis of ABA is due to the rise in abiotic stress, which, in turn, plays a role in the inhibition of ABA degradation, so pre-treatment of plants with ABA makes them more tolerant to abiotic stress.

In future studies, examining the influence of ABA-induced genes and proteins on stress tolerance, under different combinations of multiple stress conditions, will provide more detailed insights into the functions of ABA in different contexts. Unravelling the roles and relative importance of all ABA-responsive genes and proteins in rice plants is a daunting task but is necessary to enhance our vision and understanding of the myriad complexities of abiotic stress responses.

## Figures and Tables

**Table 1:** Proteins differentially expressed under four different comparisons in IAC1131.

Stress Treatments Comparison	DEPs	Increased in Abundance	Decreased in Abundance
[C(+ABA)\C(−ABA)]	87	38	49
[S(+ABA)\S(−ABA)]	148	85	63
[S(−ABA)\C(−ABA)]	609	381	228
[S(+ABA)\C(+ABA)]	789	533	256

## Data Availability

Mass spectrometry proteomics data have been deposited to the ProteomeXchange Consortium [62] via the PRIDE partner repository [63], with dataset identifier PXD030428.

## Supplementary Materials

The supplementary materials are available online at https://www.mdpi.com/article/10.3390/ijms24119628/s1.
